# Radiation Tolerance in Tardigrades: Current Knowledge and Potential Applications in Medicine

**DOI:** 10.3390/cancers11091333

**Published:** 2019-09-09

**Authors:** K. Ingemar Jönsson

**Affiliations:** Department of Environmental Science and Bioscience, Kristianstad University, 291 88 Kristianstad, Sweden; ingemar.jonsson@hkr.se; Tel.: +46-44-250-3429

**Keywords:** anhydrobiosis, cancer, cryptobiosis, desiccation tolerance, DNA repair, oxidative stress, radiation tolerance, tardigrades

## Abstract

Tardigrades represent a phylum of very small aquatic animals in which many species have evolved adaptations to survive under extreme environmental conditions, such as desiccation and freezing. Studies on several species have documented that tardigrades also belong to the most radiation-tolerant animals on Earth. This paper gives an overview of our current knowledge on radiation tolerance of tardigrades, with respect to dose-responses, developmental stages, and different radiation sources. The molecular mechanisms behind radiation tolerance in tardigrades are still largely unknown, but omics studies suggest that both mechanisms related to the avoidance of DNA damage and mechanisms of DNA repair are involved. The potential of tardigrades to provide knowledge of importance for medical sciences has long been recognized, but it is not until recently that more apparent evidence of such potential has appeared. Recent studies show that stress-related tardigrade genes may be transfected to human cells and provide increased tolerance to osmotic stress and ionizing radiation. With the recent sequencing of the tardigrade genome, more studies applying tardigrade omics to relevant aspects of human medicine are expected. In particular, the cancer research field has potential to learn from studies on tardigrades about molecular mechanisms evolved to maintain genome integrity.

## 1. Introduction

Tardigrades belong to the smallest invertebrates with a body length that may reach 1 mm in the largest species, but usually is in the range of 0.25–0.50 mm. They are essentially aquatic animals, requiring to be surrounded by water in order to maintain their hydroskeleton function and for uptake of oxygen through the cuticle. They represent a specific phylum, Tardigrada, which currently includes around 1300 described species [1,2,3] from a variety of terrestrial, freshwater and marine ecosystems. Their taxonomic and phylogenetic position remains debated, but both morphological and molecular analyses usually find them closely related to the Arthropods [4], although there are studies suggesting a closer association with nematodes [5]. The genome size of tardigrades is small and variable also among species within the same family. For example, within the Hypsibidae family, genome size of *Hypsibius exemplaris* (previously named *Hypsibius dujardini* [6]) was estimated as 104 Mb and of *Ramazzottius varieornatus* as 55 Mb [5]. These are also the first tardigrades to have their genomes sequenced [7,8].

Although the tardigrade phylum includes species with a variety of morphological and physiological adaptations, they are most well-known for the taxa that have evolved adaptations to survive dry and cold environmental conditions [9,10]. Tardigrades represent one of three invertebrate groups, including also nematodes and rotifers, where adaptations for complete desiccation and freezing of the body in both egg, juvenile and adult stages are common, allowing these populations to persist in very harsh ecosystems and microhabitats. Severe desiccation and freezing result in metabolic arrest and a state called cryptobiosis (hidden life) [11]. Cryptobiosis induced by desiccation is referred to as anhydrobiosis while cryptobiosis induced by freezing is called cryobiosis. Similar adaptations are found also in a few arthropods, but then restricted to early developmental stages (embryos, larvae) [12,13]. The tolerance of tardigrades has been known since the 1700s, but received renewed attention with the 1959 review by David Keilin [11], in which the concept of cryptobiosis was introduced. Since then, cryptobiotic organisms have been subject to studies not only for the remarkable biological achievement to desiccate completely and enter an ametabolic state, but also for its potential importance in some applied sciences where the preservation of biological material in a dry state is central [14,15].

Studies on cryptobiotic invertebrates, including tardigrades [16], bdelloid rotifers [17], the crustacean *Artemia salina* [18], and the chironomid *Polypedilum vanderplanki* [19], have shown that they are also highly tolerant to radiation. Also, in strains of prokaryotes, radiation tolerance has been reported to correlate with desiccation tolerance [20], although a recent study on anaerobic bacteria did not find support for such a correlation [21]. Cross-tolerance between desiccation and radiation may originate from a common underlying adaptive protection mechanism that is activated not only against the natural environmental agents but also against other agents that give rise to similar cellular stress, for example, radiation [20,22,23]. This review gives a brief overview of our knowledge on radiation tolerance in tardigrades, the possible molecular mechanisms behind the tolerance, and the potential importance of tardigrade tolerance research to medical sciences with an emphasis on cancer.

## 2. The Patterns of Tolerance to Radiation in Tardigrades

### 2.1. Low Linear Energy Transfer (Low-LET) Irradiation

**Figure 1:**
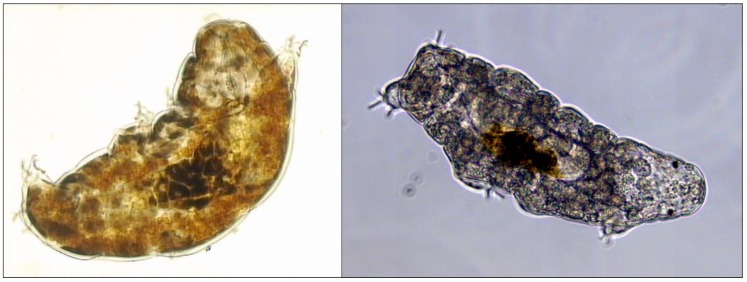
Light microscope images of two of the tardigrade species that have been used in studies on radiation tolerance, *Richtersius* cf. *coronifer* (**left panel**) and *Hypsibius exemplaris* (**right panel**).

**Figure 2:**
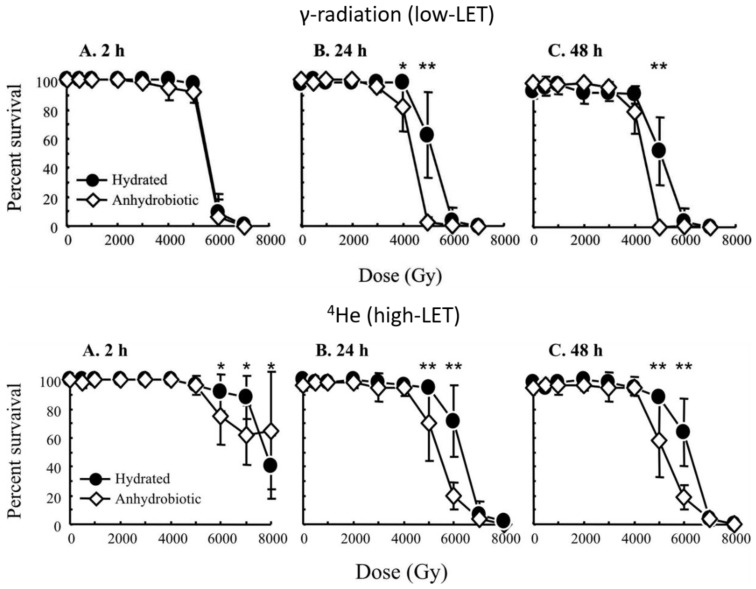
Dose-responses of the eutardigrade *Milnesium tardigradum* to exposure of γ-radiation (low-LET, upper panel) and helium ions (4He, high-LET, lower panel). Survival estimates after irradiation of both hydrated and desiccated (anhydrobiotic) animals are shown, and for estimates at 2, 24, and 48 h postirradiation (hydrated animals) or postrehydration (desiccated animals). Reprinted from Horikawa et al. [27] (Figure 1 and Figure 2) by permission of the publisher (Taylor & Francis Ltd.). Asterisks represent significant differences in survival between the hydrated and anhydrobiotic groups at *p* < 0.05 (*) and *p* < 0.001 (**) levels.

The tolerance of tardigrades to severe desiccation was recognized soon after this animal group was discovered and described in the 1770s [24], while the first scientific study on radiation tolerance in tardigrades was reported by May et al. in 1964 [25], where adult animals of the species *Paramacrobiotus areolatus* were irradiated by X-ray and ultraviolet (UV) radiation both in the desiccated state and in the hydrated active state. The results for X-ray showed a very high tolerance, with similar dose responses in the desiccated and hydrated states, and LD50_24h_-values between 5 and 6 kGy. Later studies on other semi-terrestrial or freshwater tardigrade species (*Milnesium tardigradum, Richtersius* cf. *coronifer*, *H. exemplaris*; Figure 1 and Figure 2) using γ-ray have given similar results, with estimates of LD50 between 3 and 5 kGy [26,27,28]. More detailed summaries of dose-response studies in tardigrades can be found elsewhere [16,29]. In the only marine species investigated, *Echiniscoides sigismundi*, tolerance to γ-ray was clearly lower, with an LD50 estimate around 1.5 kGy 7 days post-irradiation [30]. The similar dose-response in desiccated and hydrated tardigrades (Figure 2) seems to be a general feature, which is unexpected given that the indirect effects of ionizing radiation are expected to be much higher in the presence of water [31]. X-ray and γ-ray represent low-LET radiation, with biological effects relying mainly on indirect radiation effects through the creation of reactive oxygen species (ROS) that damage cell components such as DNA and proteins. One study on bystander effects of radiation has been reported [32], where tardigrades were irradiated with γ-ray (3 and 5 kGy) and single irradiated individuals were transferred to groups of unirradiated animals that were monitored for survival up to 30 days. A bystander effect (reduced survival) was observed in comparison to controls, but there was no difference between the two irradiation doses, and thus no indication of a dose-response.

### 2.2. High Linear Energy Transfer (High-LET) Irradiation

A number of studies using high-LET radiation on tardigrades have also been reported. The biological effect of high-LET radiation comes mainly from direct impacts and deposition of energy in the tissue and cells. The higher biological impact of direct hits in the case of high-LET radiation also means that the importance of chemical protectors (e.g., ROS scavengers) is expected to be lower compared to low-LET radiation. Exposures of adult tardigrades to high-LET radiation have included protons [33], helium ions [27,34], and iron ions [34]. These studies generally suggest that adult tardigrades are as tolerant to high-LET radiation as they are to low-LET radiation, and in some cases, even more tolerant [27,33]. For instance, in the study with protons, the value of LD50_24h_ was estimated at ca. 10 kGy in *R.* cf. *coronifer* [33]. In a study by Horikawa et al. [27] using helium ions (^4^He), both desiccated and hydrated *M. tardigradum* were tested, and at higher doses, the animals tended to survive better in the hydrated state compared to desiccated animals (Figure 2).

### 2.3. Irradiation with UV

There are few studies on tolerance to ultraviolet radiation in adult tardigrades, but those reported generally indicate a lower tolerance to UV in the hydrated state [25,35,36], although hydrated tardigrades have been reported to show higher tolerance to UV radiation than desiccated ones when irradiated under low temperature [35]. In a study by Horikawa et al. [36], two different species, *R. varieornatus* and *H. exemplaris*, were exposed to UVC doses of 2.5–20 kJm^−2^, and the results showed a clear difference in sensitivity between the species and between irradiation in the hydrated and desiccated state in *R. varieornatus* (*H. exemplaris* was not tested in the desiccated state). While *R. varieornatus* showed high survival (ca. 80% 5 days post-irradiation) at the lowest dose of 2.5 kJm^−2^ in the hydrated state, *H. exemplaris* did not survive this exposure. Irradiation in the desiccated state of *R. varieornatus* resulted in much higher survival, with around 80% of the animals exposed to 20 kJm^−2^ being alive 13 days after the irradiation, but thereafter, survival declined more rapidly in this group compared to lower doses. In 2007, desiccated tardigrades of the species *R.* cf. *coronifer* and *M. tardigradum* were exposed in space during 10 days to the combined effect of space vacuum (10^−6^ Pa), cosmic radiation (100 mGy) and UV radiation of two different spectra (UV_A_+_B_: 280–400 nm, total dose 7095 kJm^−2^; UV_vacuum_ to UV_A_: 116.5–400 nm, total dose 7577 kJm^−2^) at low Earth orbit within the Tardigrades in Space (TARDIS) project [37,38]. Compared to samples protected from solar light, which were not affected significantly by the exposure to cosmic radiation and space vacuum, UV radiation-exposed tardigrades suffered a high mortality, and no animals of the two species survived the full UV spectrum. However, 12% of the *M. tardigradum* specimens exposed to UV_A_+_B_ survived, representing the first animals ever to survive the combined exposure of vacuum, cosmic radiation, and UV radiation under space conditions [37].

### 2.4. Effects on Fertility and Hatchability of Laid Eggs

In dose-response studies where adult tardigrades were irradiated with low- and high-LET radiation, egg production and the viability of laid eggs were affected at much lower doses than the adults, with few eggs laid and hatched above a dose of 100 Gy [26,27,28]. This is also expected since embryos represent developing tissues with a higher proliferation rate and, therefore, generally are expected to be more vulnerable to radiation [39]. In *R.* cf. *coronifer* irradiated in a hydrated state, egg production was normal up to 1 kGy but then declined rapidly to 4% of normal production at 4 kGy, and no irradiated eggs hatched [26]. Other studies suggest that the main effect of radiation is on fertility rather than on the viability of produced eggs. In the UVC study by Horikawa et al. [36], fertility was more affected by radiation than survival of the animals, but hatching of the laid eggs did not differ among treatment groups. The same was observed in the TARDIS space experiment, where exposure to UV had a strong effect on survival and egg production, but not on the hatchability of laid eggs or fertility of descendant generations [37,38]. This indicates that effects of radiation on reproductive functions may be manifested mainly through reduced egg production and that the development of damaged eggs is terminated before laying while only undamaged eggs are laid, but more studies are needed to evaluate this hypothesis.

### 2.5. Direct Irradiation of Early Developmental Stages

**Figure 3:**
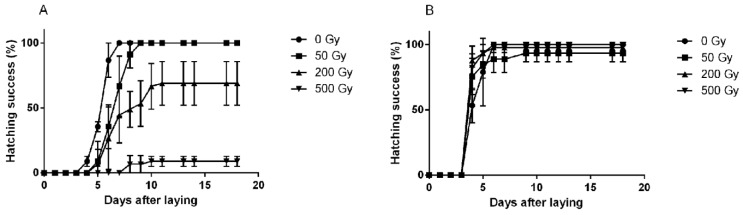
Patterns of hatching success of *Hypsibius exemplaris* eggs irradiated in the hydrated state with different doses of γ-ray and at two different stages of egg development; (**A**) early developmental stage and (**B**) late developmental stage. The error bars represent standard deviations from three repeats, each with 15 eggs. Reprinted from Beltrán-Pardo et al. [28] (Figure 3) under a Creative Commons Attribution-NonCommercial-ShareAlike 4.0 International License.

A few studies using low-LET radiation have investigated the tolerance of tardigrade embryos to direct irradiation. Studies in three different tardigrade species (*R.* cf. *coronifer*, *Milnesium* cf. *tardigradum*, *H. exemplaris*) have found a similar pattern of radiation sensitivity with respect to developmental stage, with the early stage being very sensitive, while in the late stage no effect of radiation up to 500 Gy (which was the highest dose used) was observed [28,40,41]. In two of these studies [28,40], a clear dose-response was observed in the early developmental stage, both with respect to the final hatching success and with respect to the initiation and rate of hatching (Figure 3). Thus, irradiation in the early developmental stage resulted in lower hatching success and delayed hatching. Two possible reasons for this change in sensitivity to radiation over the developmental period may be a higher frequency of cell division in the early stage of development or that cellular protection mechanisms are not developed and activated until the mid- or late stage of development. Both alternatives may contribute to the observed higher sensitivity in the early developmental stage, but no further studies evaluating these possible factors have been reported. It is noteworthy that a similar change in sensitivity over the course of egg development has been reported with respect to desiccation [42].

Only one study has evaluated the tolerance of tardigrade embryos to high-LET radiation (^4^He, *R. varieornatus*) [43]. The embryos in this study were irradiated in the mid-stage of development. In contrast to irradiation studies on adults, hydrated embryos showed a clearly higher sensitivity than desiccated embryos, with LD50 estimates (based on hatchability) for hydrated embryos at 509 Gy and for desiccated embryos at 1690 Gy. Increased sensitivity to radiation (γ-ray) with higher hydration levels has also been reported in cysts of *A. salina* [44], and larvae of *P. vanderplanki* show a higher sensitivity to both low-LET (γ-ray) and high-LET (^4^He) radiation in the fully hydrated state, compared to desiccated larvae [45,46].

In the 2007 TARDIS space experiment, eggs of *R.* cf. *coronifer* and *M. tardigradum* were directly exposed to space vacuum, cosmic radiation and UV radiation. No eggs hatched when exposed to these factors simultaneously, while exposure to space vacuum + cosmic radiation had no significant effect on hatchability [37].

## 3. Molecular Mechanisms of Radiation and Desiccation Tolerance in Tardigrades

### 3.1. Damage Induced by Radiation

Despite a considerable amount of studies documenting phenotypic dose-responses to radiation in tardigrades, studies on cellular and molecular damage induced by radiation are very scarce. There are currently no studies documenting the amount of damage to DNA from low-LET or high-LET radiation in tardigrades, but two studies have reported DNA damage after UV radiation. Horikawa et al. [36] analyzed the induction of thymidine dimers from UVC radiation (254 nm) and showed a strong dose-dependent increase in dimer formation in the two species studied (*R. varieornatus*, *H. exemplaris*) after irradiation in the hydrated state, while dimer formation after irradiation in the desiccated state (*R. varieornatus* only) was much less. In *R. varieornatus*, but not in the more desiccation-sensitive species *H. exemplaris*, the frequency of dimers declined after the initial increase and had returned to the control levels after 112 h, indicating a long repair process.

In another study, comet assay was used to analyze DNA damage in desiccated cells of the tardigrade *M. tardigradum*, and UVB (312 nm) at a dose of 3.735 Jm^−2^ was used as positive control [47]. This irradiation led to significant fragmentation of DNA (ca. 17% DNA in comet tail, compared to 0.44% in controls), but did not affect the survival or reproduction of the tardigrades. The repair process of this UV-induced damage was not reported. One study on protein carbonylation, damage to proteins originating from reactive oxygen species (ROS), in tardigrades, has been reported [48]. Carbonylation is a common marker of oxidative stress [49], and the study showed increased accumulation of carbonylation with higher doses of UVC (60–180 kJm^−2^) and also that carbonylation tended to increase with time spent in the anhydrobiotic state (up to 73 days).

### 3.2. Molecular Responses to Radiation

There are few studies specifically related to the molecular system involved in responses to radiation in tardigrades. Most omics studies have had a focus on documenting molecular responses to desiccation and identifying genes and pathways activated during anhydrobiosis [50,51,52,53]. One of the exceptions is the study by Beltran-Pardo et al. [54], showing a strong upregulation of the Rad51 protein in hydrated active *M.* cf. *tardigradum* after γ-irradiation (70 Gy), suggesting an activation of homologous DNA repair pathways in response to radiation damage. In another study, Jönsson & Schill [55] analyzed the expression of the stress protein Hsp70 after desiccation and irradiation (of both desiccated and hydrated animals) with γ-ray (500 Gy) in *R.* cf. *coronifer*. Expression of Hsp70 was upregulated after both desiccation and radiation and significantly more so in the irradiated animals, with highest levels in the group irradiated in the hydrated state. Hsp70 is a family of highly conservative heat-shock proteins with many functions related to stress response and maintaining of genomic stability, for example, chaperone protection of proteins and prevention of apoptosis, and with relevance to many diseases including cancer [56,57].

**Figure 4:**
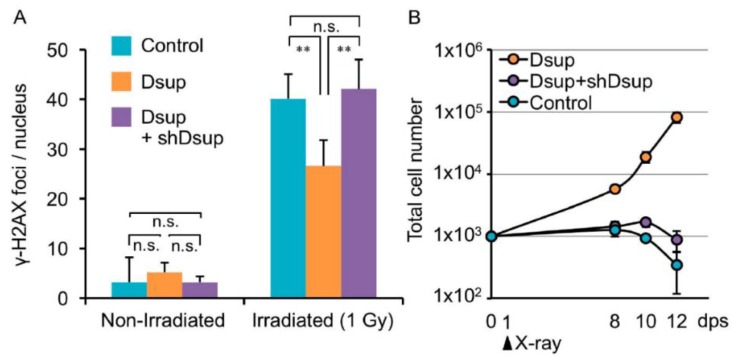
Effects of expression of the tardigrade gene Dsup in human cells (HEK293) on DNA strand breaks (**A**) and cell viability (**B**) after irradiation with X-ray. The figures show the results for non-transfected cells (Control), transfected cells expressing the gene (Dsup), and transfected cells where the gene was not expressed (Dsup+shDsup) (**A**). Estimates of DNA strand breaks using the marker γ-H2AX of non-irradiated and X-ray irradiated (1 Gy) cells. (**B**). Growth patterns of cells of the three treatment groups up to 12 days post-seeding (dps) after X-ray irradiation (4 Gy). Reprinted from Hashimoto & Kunieda [29] (Figure 2) under a Creative Commons Attribution-NonCommercial-ShareAlike 4.0 International License.

In a study on the desiccation and radiation tolerant tardigrade *R. varieornatus*, Hashimoto et al. [8,29] reported a tardigrade-unique protein, Damage supressor (Dsup), that associated with nuclear DNA and therefore potentially could play a role in protecting DNA from damage. Transfecting the gene for Dsup into human embryonic kidney cells (HEK293) resulted in improved viability and a reduction of DNA damage by up to 40% after irradiation with X-rays, compared to irradiated non-transfected cells (Figure 4). The reduced damage to DNA suggests that Dsup has a function of protecting DNA from strand breaks, but the details of such function remain unknown.

### 3.3. Molecular Mechanisms of Desiccation Tolerance

Considerably more studies have been reported on molecular responses to desiccation in tardigrades than to radiation. This is not surprising since the importance of understanding how desiccation-tolerant animals such as tardigrades are able preserve their cells under complete dehydration has been recognized for a long time [11,58,59]. This was also the focus in the early period of modern studies on cryptobiosis, beginning in the early 1970s. Given that cells under anhydrobiosis lack metabolism and, therefore, essential dynamics, their potential for returning to an active metabolic state is strongly connected to mechanisms of structural integrity. Studies on how membranes are stabilized in the dry state were developed within the ”water replacement” framework, with a focus on the role of the disaccharide trehalose as membrane stabilizer [15,60]. This framework was later complemented by the ”vitrification” hypothesis, still with focus on trehalose as a molecular compound that forms a glassy state under dry conditions [61]. In contrast to some other invertebrate groups with anhydrobiosis where trehalose levels in the dry state reach 15–20% of dry weight (nematodes, *Artemia*, *P. vanderplanki*), tardigrades have much lower levels (below 3%), and only in one family (Macrobiotidae) is the level upregulated in response to desiccation [62,63,64]. However, evidence of vitrification in desiccated tardigrades has been reported, and interestingly, the species where glass transition and a glassy state were documented all belong to the Macrobiotidae family where trehalose is induced [9,65]. Although this indicates a possible involvement of trehalose, whether this disaccharide is in fact contributing to the observed vitrification and is part of the protection system of tardigrades will require further studies. A study in *H. exemplaris* (family Hypsibidae) reported evidence that also this species enters a vitrified state during desiccation, and that one group (Cytosolic Abundant Heat Soluble, CAHS) of identified tardigrade-specific intrinsically disordered proteins (TDP) with vitrification properties is induced during dehydration and necessary for tolerating desiccation [53]. Since *H. exemplaris* does not synthesize trehalose, this indicates convergent evolution in different tardigrade taxa towards similar protection mechanisms but based on different molecular pathways. In the same study, the CAHS gene was also transfected to bacteria (*Escherichia coli*) and yeast, and shown to improve their tolerance to desiccation [53].

In the recent decade, supported by the rapidly developing omics fields, more broad-scale molecular analyses of genes and pathways associated with stress responses have emerged, with the aim to reveal the ”desiccome” [66] of anhydrobiotic tardigrades. The identification of the group of intrinsically-disordered TDP proteins mentioned above, which includes several different families of heat-soluble proteins with different subcellular localizations (CAHS; Secreted Abundant Heat Soluble, SAHS; Mitochondrial Abundant Heat Soluble, MAHS; Ramazzottius varieornatus Late Embryogenesis Abundant Mitochondrial, RvLEAM) represents an important outcome of these efforts [67,68]. These proteins have been found in several species of the Eutardigrada class, but were recently reported to be missing in the first species of the Heterotardigrada class (*E. sigismundi*) that has been subject to transcriptomic analysis [69]. The tardigrade omics studies have also led to an increased interest in and knowledge about the role of oxidative stress-protection mechanisms and DNA repair in tardigrades. Desiccation gives rise to ROS [70], and the antioxidant systems evolved in cryptobiotic animals to handle desiccation stress are candidates to explain cross-tolerance between desiccation and radiation. Several studies based on genomics, transcriptomics and proteomics have documented the expression of a wide variety of known antioxidants in desiccated tardigrades compared to hydrated ones [29,51,69,71,72,73]. These omics studies have also provided information on the expression of a number of DNA repair pathways (e.g., Mre11, DNA repair endonuclease XPF, ubiquitin, DnaJ family, Rhp57, proteasome maturation factor, mutS, POLE) [5,8,50,52,69,72], suggesting the presence in tardigrades of all major pathways of DNA repair (non-homologous end joining, homologous recombination, mismatch repair, nucleotide excision repair, base excision repair). An exception seems to be the marine heterotardigrade *E. sigismundi*, which was reported to lack pathways for non-homologous end joining (c-NHEJ), one of the major pathways for repair of DNA double-strand breaks besides homologous recombination (HR) [69]. This species is tolerant to rapid desiccation [74], but seems to be less tolerant to long-term anhydrobiosis compared to semi-terrestrial tardigrades [75], and as mentioned above, it also has a lower tolerance to ionizing radiation [30]. Whether the lower radiation tolerance in *E. sigismundi* is related to the observed difference in DNA repair pathways remains to be investigated.

An emerging picture from the omics studies of tardigrades is a variation in the molecular stress response among tardigrade taxa, both with respect to the expression of specific genes and with respect to whether the response system is induced or constitutive. Species tolerating rapid dehydration (*R. varieornatus*, *M. tardigradum*) seem to rely more on a constitutively-expressed protection system, while more dehydration-sensitive species (*H. exemplaris*, *Paramacrobiotus richtersi*) show more induced expression of the protection pathways [5,53]. These differences likely represent adaptations to the different environmental conditions under which the species have evolved.

### 3.4. Cross-Tolerance between Desiccation and Radiation

Tolerance to radiation in desiccation-tolerant organisms has usually been explained as a cross-tolerance phenomenon originating from activation of the same cell-protection system that has evolved to cope with desiccation-induced stress and damage. No studies on tardigrades have so far evaluated this hypothesis specifically, neither with respect to correlative patterns in survival or with respect to expressions of molecular pathways when exposed to desiccation and radiation, respectively. Several species that are tolerant to rapid desiccation also have a high radiation tolerance (*R.* cf. *coronifer*, *M. tardigradum*, *R. varieornatus*) [26,27,36,76,77,78], but *H. exemplaris* and *E. sigismundi* seem to deviate from this pattern, but in different ways. *H. exemplaris* is sensitive to rapid desiccation but tolerate high ionizing radiation [28,36,77], while *E. sigismundi* tolerates rapid desiccation but is more sensitive to ionizing radiation [30,74]. For a more comprehensive picture of how desiccation and radiation tolerance are connected, studies on more species that provide a broader range of tolerances will be needed, and particularly important will be species that do not tolerate desiccation. No studies on radiation tolerance in tardigrades without cryptobiotic capabilities have so far been reported.

In larvae of the chironomid *P. vanderplanki*, Gusev et al. [79] reported upregulation of genes for DNA repair (Rad23, Rad51) after both desiccation and radiation (^4^He, γ-ray), and for antioxidants (catalase, Cu/Zn-superoxide dismutase, glutathione peroxidase) after desiccation and γ-radiation, supporting the cross-tolerance hypothesis. In another attempt to evaluate the cross-tolerance hypothesis at the genetic level in *P. vanderplanki*, Ryabova et al. [80] analyzed the transcriptional response to both desiccation, low-LET radiation (γ-ray), and high-LET radiation (^4^He). They found a broader transcriptional expression in response to desiccation compared to radiation, but also an overlap in the expression of some anhydrobiosis-related groups, including antioxidants, late embryogenesis-abundant (LEA) proteins, and heat-shock proteins (Hsp). The results of this study were interpreted as support for a common molecular response underlying the observed high tolerance to desiccation and radiation in *P. vanderplanki*, representing an evolved adaptive mechanism to survive desiccation. Similar omics analyses in tardigrades would be welcome, and the inclusion of several species with different profiles of desiccation and radiation tolerance in such analyses would be of particular interest.

## 4. Relevance of Tardigrade Stress Mechanisms for Research on Cancer and Other Medical Fields

### 4.1. Preservation of Cells, Tissues and Bioreagents in the Dry State

In the course of evolution, tardigrades and other cryptobiotic animals have evolved solutions to a number of challenging biological conditions that should be of considerable interest to medicine. The potential importance of knowledge on cryptobiotic organisms for the medical fields was recognized already by Keilin [11] in his 1959 review, where he mentioned “resistance of microorganisms, of their spores and cysts to desiccation, their longevity and their dissemination in nature, which is of great medical and agricultural importance”, “The preservation of biological materials, such as tissues for transplantations, blood for transfusion, semen for artificial insemination, micro-organisms for type cultures, seeds for germination and certain articles of food”, and “Hypothermia, or lowering the temperature and therefore the metabolism of mammals and man, which is of great biological interest and of medical as well as surgical importance” (p. 165). This view on cryptobiotic organisms has followed the development of research ever since, and the work on membrane systems and dehydration soon gave rise to applications in the medical field, including preservation of liposomes [81] and blood platelets [82,83] using trehalose. Crowe [15] has also reviewed some other medical applications of trehalose related to osteoporosis, Huntington’s Disease, and autophagy. As new molecular compounds with similar protection characteristics as trehalose are found in tardigrades, similar applications in medical science may be expected. One family of the tardigrade-specific intrinsically-disordered proteins (TDPs), cytosolic abundant heat-soluble (CAHS) proteins, has recently been tested for stabilization of pharmaceutical excipients (lactate dehydrogenase, lipoprotein lipase) with promising results [84].

### 4.2. Cell and Genomic Integrity—A Field of Common Interest in Research on Tardigrades and Cancer

How genome integrity is maintained is a central aspect in understanding radiation tolerance of tardigrades and other cryptobiotic organisms [16,29], and so also in the fields of human carcinogenesis and radiotherapy [85,86]. Both mechanisms that prevent damage to DNA (e.g., ROS scavengers) and repair damage to DNA are likely involved in the desiccation/radiation tolerance of tardigrades, and the emerging understanding of these mechanisms should be of interest to cancer research. The reported transfection of the Dsup gene into human embryonic kidney cells (HEK293) resulting in increased tolerance to radiation [8] should be an eye-opener to the potential benefits of collaboration between medical research and research on cryptobiotic animals.

Oxidative stress is a field where studies on the tolerance of tardigrades have a high potential to contribute to medical research. Oxidative stress is a central aspect in the development of many diseases, including, for example, cancer, aging, diabetes, inflammation, and Parkinson’s disease [87]. It is also an important aspect of radiotherapy, since the effect of low-LET radiation treatment of cancer cells primarily relies on increased oxidative stress created by radiation-induced ROS. The production of ROS is an important consequence of both desiccation [70] and ionizing radiation, and as indicated above, the antioxidant system plays a central role in the tolerance to desiccation (supposedly also to radiation) in cryptobiotic animals [88]. The documented effect of the tardigrade-specific protein Dsup in reducing radiation damage to DNA in human HEK293 cells was also shown be connected to reduced impact of ROS by hydrogen peroxide [8]. In another study, also in the tardigrade *R. varieornatus*, genes for the mitochondrial TDP proteins RvLEAM and MAHS, proposed to be involved in the protection of mitochondria from desiccation stress, were transfected to human cells (HEp-2) and shown to improve hyperosmotic tolerance of the human cells [68]. To my knowledge, the transfections of Dsup and RvLEAM/MAHS to human cells represent the first transfers of genes encoding (assumed) protective proteins from tardigrades to human cells. The positive results of these studies indicate the potential for applying the molecular protection system of tardigrades into medical sciences, in particular, cancer research. The now available genomics, transcriptomics, and proteomics data from tardigrades also provide a rich material for more detailed studies on the antioxidant and DNA repair system in tardigrades and for experimental studies where tardigrade genes are tested in human cells.

Most studies on radiation tolerance in tardigrades have aimed to document dose-responses with very high doses and survival and (in some cases) fertility as endpoints [25,26,27,28,30,33,34]. Exposures of tardigrades to lower non-lethal doses and experimental studies more directly related to the central fields of human cancer and radiotherapy may be an important step towards making this research more relevant to cancer research. This may include studies on, for example, effects of dose rate, fractionation, hypoxia, and bystander effects, as well as cell biology responses to radiation such as cell cycle arrest, apoptosis, senescence, and autophagy. These are aspects where hardly any research has yet been reported in tardigrades, but which are central to current cancer research.

## 5. Conclusions

Studies over the last 15 years have documented a very high tolerance to both low-LET and high-LET radiation in adult tardigrades, with estimates of LD50 for short-term survival in the range of 1.5–10 kGy, depending on species and radiation source. Fertility and developing embryos are generally much more sensitive than adults, and embryos are particularly sensitive in the early stage of development. In studies where both desiccated and hydrated animals have been irradiated with low- or high-LET radiation, the dose-responses are similar, showing that tolerance is not restricted to protection mechanisms in the dry state but relying on mechanisms in the metabolically-active animals. Radiation tolerance in cryptobiotic animals are usually explained as a cross-tolerance phenomenon, representing a by-product of adaptive protection mechanisms to survive desiccation. No studies have so far provided direct molecular evidence for this in tardigrades, although it is the most plausible explanation. Antioxidant and DNA-repair pathways, evolved to protect the cells from oxidative stress and damage to proteins, lipids and DNA in connection with dehydration, are likely components of such protection systems underlying radiation tolerance. Over the last decade, our knowledge on the molecular stress-response systems in tardigrades has increased considerably from a number of genomics, transcriptomics and proteomics studies, and several new proteins unique to tardigrades have been identified. These studies have mainly focused on expression patterns related to desiccation, and more omics studies analyzing molecular responses to radiation are necessary in order to understand radiation tolerance in tardigrades. We also need studies documenting the extent to which radiation leads to DNA damage and the kinetics of repair.

Research on the mechanisms of tolerance to desiccation and radiation in tardigrades has a large potential to contribute interesting knowledge to the medical fields, including cancer. Just as the model organisms *Caenorhabditis elegans* and *Drosophila melanogaster* for many years have played important roles in the development of our understanding of cancer [89,90], the molecular biology of cryptobiotic invertebrates such as tardigrades should have much insights to offer cancer research on mechanisms related to genetic integrity and cell protection, especially antioxidative responses and DNA repair. Belonging to the same superclade as *C. elegans* and *D. melanogaster*, Ecdysozoa (molting animals), but representing an animal with adaptations to much more challenging cellular conditions, tardigrades provide opportunities for comparative analyses with the other model organisms and with human cells in the field of cancer. This will take advantage of the variety of molecular solutions to cellular challenges that have evolved in different organisms.
